# Predictive Usefulness of Urinary Biomarkers for the Identification of Cyclosporine A-Induced Nephrotoxicity in a Rat Model

**DOI:** 10.1371/journal.pone.0103660

**Published:** 2014-07-29

**Authors:** Carla Patrícia Carlos, Nathália Martins Sonehara, Sonia Maria Oliani, Emmanuel A. Burdmann

**Affiliations:** 1 Division of Nephrology, São José do Rio Preto Medical School, São José do Rio Preto, SP, Brazil; 2 Department of Biology, Instituto de Biociências, Letras e Ciências Exatas, São Paulo State University, São José do Rio Preto, SP, Brazil; 3 LIM 12, Division of Nephrology, University of São Paulo Medical School, São Paulo, SP, Brazil; National Institutes of Health, United States of America

## Abstract

The main side effect of cyclosporine A (CsA), a widely used immunosuppressive drug, is nephrotoxicity. Early detection of CsA-induced acute nephrotoxicity is essential for stop or minimize kidney injury, and timely detection of chronic nephrotoxicity is critical for halting the drug and preventing irreversible kidney injury. This study aimed to identify urinary biomarkers for the detection of CsA-induced nephrotoxicity. We allocated salt-depleted rats to receive CsA or vehicle for 7, 14 or 21 days and evaluated renal function and hemodynamics, microalbuminuria, renal macrophage infiltration, tubulointerstitial fibrosis and renal tissue and urinary biomarkers for kidney injury. Kidney injury molecule-1 (KIM-1), tumor necrosis factor-alpha (TNF-α), interleukin 6 (IL-6), fibronectin, neutrophil gelatinase-associated lipocalin (NGAL), TGF-β, osteopontin, and podocin were assessed in urine. TNF-α, IL-6, fibronectin, osteopontin, TGF-β, collagen IV, alpha smooth muscle actin (α -SMA) and vimentin were assessed in renal tissue. CsA caused early functional renal dysfunction and microalbuminuria, followed by macrophage infiltration and late tubulointerstitial fibrosis. Urinary TNF-α, KIM-1 and fibronectin increased in the early phase, and urinary TGF-β and osteopontin increased in the late phase of CsA nephrotoxicity. Urinary biomarkers correlated consistently with renal tissue cytokine expression. In conclusion, early increases in urinary KIM-1, TNF-α, and fibronectin and elevated microalbuminuria indicate acute CsA nephrotoxicity. Late increases in urinary osteopontin and TGF-β indicate chronic CsA nephrotoxicity. These urinary kidney injury biomarkers correlated well with the renal tissue expression of injury markers and with the temporal development of CsA nephrotoxicity.

## Introduction

The main side effect of CsA, a widely used immunosuppressant agent, is nephrotoxicity, which can be manifested as acute or chronic. Acute nephrotoxicity is a functional and reversible phenomenon, whereas chronic nephrotoxicity is characterized by irreversible interstitial fibrosis, which usually leads to impaired renal function [1].

The therapeutic window for CsA is narrow and variable, and CsA blood levels are little help in predicting kidney injury development. CsA-induced nephrotoxicity is usually diagnosed only when serum creatinine increases, denoting a significant impairment of the glomerular filtration rate (GFR). The line between a functional reversible injury and irreversible chronic tubulointerstitial fibrosis is inexact. In fact, significant structural injury can occur in the presence of normal or minimally altered serum creatinine levels [2]. The identification of biomarkers that timely detects the initiation of CsA nephrotoxicity and enable its progression to be monitored would be extremely useful, allowing maneuvers to halt or minimize kidney injury progression.

Several urinary biomarkers for the detection of kidney injury have been tested [3]–[4], but few studies have evaluated urinary biomarkers specific for diagnosing CsA-induced nephrotoxicity.

The aim of this study was to investigate urinary biomarkers for the identification of both acute and chronic CsA nephrotoxicity and to correlate them with the development of functional and structural changes in a rat model of CsA-induced nephrotoxicity. Those urinary biomarkers included kidney injury molecule-1 (KIM-1) and neutrophil gelatinase-associated lipocalin (NGAL) [5]–[8]; podocin [9]–[10]; osteopontin, tumor necrosis factor-alpha (TNF-α), interleukin 6 (IL-6) and microalbuminuria [11]–[18]; and TGF-β, alpha-smooth muscle actin (α-SMA), collagen and fibronectin [19]–[21].

## Materials and Methods

### Study Design

The Ethical Animal Experimentation Committee of São José do Rio Preto Medical School, Brazil, approved the protocol and all the procedures (process 3992/2009).

Groups of eight male Munich-Wistar rats weighing approximately 250 g (São José do Rio Preto Medical School) were randomly assigned to receive CsA (treatment group) or vehicle (control group, VH). CsA powder was dissolved in VH (12.5% ethanol and 87.5% olive oil) to a final concentration of 15 mg/mL. The treatment group received a daily subcutaneous injection of CsA (15 mg/kg/day) for 7, 14 or 21 days. The control group received a daily subcutaneous injection of VH (1 mL/kg body weight) for 7, 14 or 21 days.

The animals received a low-salt diet of cooked rice (0.05% sodium, 8.5% protein, 76.6% carbohydrate, less than 4.3% fat) combined with amino acid supplements (Aminomix Pet; Vetnil Ind. Com. Prod. Veterinários Ltda; Louveira, SP, Brazil), starting one week before treatment and continuing throughout the study. Dietary consumption and weight gain were verified on a daily basis. Control animals were pair-fed with CsA-treated rats. The animals were allowed ad libitum access to water.

After 7, 14 and 21 days of treatment, rats were placed in metabolic cages (Nalgene, NY, USA). Their urine volume was collected and measured for 24 hours, and urine samples were taken at the final of this period.

The animals were then submitted to renal function studies, including GFR and renal blood flow (RBF). At the end of the renal function experiments, blood samples (3 mL) were drawn, the animals were euthanized with intravenously injected thiopental (100 mg/kg body weight), and renal tissue samples were collected.

### Renal Function Studies

GFR and RBF assessments were performed 24 h after the last injection of CsA or VH. After anesthesia with intraperitoneal thiopental (50 mg/kg body weight), tracheotomy was performed, and polyethylene tubes (PE-50) were placed in the carotid artery to monitor the mean arterial pressure and in the jugular vein to collect blood and perform infusions. A PE 190 polyethylene tube was inserted in the urinary bladder through a lower median abdominal incision for urine collection. Next, a ventral midline incision was made, exposing the renal artery. A suitable probe (R series, 1.5 mm, with J reflector; Transonic Systems Inc., Ithaca, NY, USA) was placed around the renal artery. A loading injection of 5 mL of 0.9% NaCl was administered, and each animal received a loading dose of 1 mL of inulin (Sigma Chemical Co., St. Louis, MO, USA) diluted in 0.9% NaCl (1 mg/mL), followed by a maintenance infusion of the same solution at 0.06 mL/min. After a 50-min equilibration, urine was collected in previously weighed vials during three 20-min periods. A blood sample (0.3 mL) was drawn at the midpoint of each urine collection and was replaced with an equal volume of 0.9% NaCl. This approach for volume infusion (loading dose, rate of infusion pump and replacements after blood collection) has been shown previously to replace adequately volume losses during experiment. Serum and urine inulin levels were measured using a colorimetric assay (anthrone method, BTS 310 spectrophotometer; BioSystems, Barcelona, Spain). The inulin clearance values, expressed as mL/min/100 g body weight, are presented as the mean of the three clearance periods. The MAP results are presented as the mean of all the recordings during the MAP period. Ultrasonic transit-time RBF measurements were taken (T 106; Transonic Systems Inc., Ithaca, NY, USA) during a 60-min observation period (measured over six points at 10-min intervals). MAP measurements were taken concurrently, and the renal vascular resistance (RVR) was calculated. RBF and RVR are presented as the mean of the six observation periods.

### Markers in Urine and Blood Samples

#### Electrolytes, Creatinine, Osmolality, Microalbuminuria, Cyclosporine, Hematocrit

Sodium and potassium levels were determined using an electrolyte analyzer (Mod. 9180; AVL Scientific Co., Roswell, GA, USA), and creatinine levels were ascertained with a colorimetric assay (BTS 310 spectrophotometer; BioSystems, Barcelona, Spain) in the 24-hour urine and in the blood samples taken after the renal function studies. Urinary osmolality was determined by freezing point depression (Osmette A; Precision Systems Inc., Natick, MA, USA) in the 24-hour urine. Microalbuminuria was assessed in the 24-hour urine with a colorimetric assay (BTS 310 spectrophotometer; BioSystems, Barcelona, Spain). CsA blood levels were analyzed by double antibody radioimmunoassay (CYCLO-Trac SP; DiaSorin, Stillwater, MN, USA), and hematocrit was assessed by the microhematocrit method in the blood samples taken after the renal function studies.

### Urinary Biomarkers for CsA Nephrotoxicity

The biomarkers were quantified by ELISA at the end of each time period (7, 14 and 21 days) using the 24-hour urine.

KIM-1 (RKM 100, R&D Systems, Minneapolis, USA) was used to identify proximal tubular and acute kidney injury. NGAL (KIT 046, BioPorto, Denmark) was used as a tubular and acute kidney injury biomarker. Fibronectin (KT-415, Kamiya, WA, USA) and TGF-β (G7590, Promega, WI, USA) were used as fibrosis biomarkers. Osteopontin (ADI-900-090A, Enzo, PA, USA), TNF-α (CKR041, Cell Sciences, MA, USA) and IL-6 (DY506, R&D Systems, Minneapolis, USA) were used as markers of macrophage infiltration and inflammation, and podocin (E90938Ra, Uscn Life Science Inc., P.R. China) served as a glomerular damage biomarker.

### Markers in Tissue Samples

#### Histopathological Analysis (Glomerular Injury and Tubulointerstitial Fibrosis)

Renal fragments collected at the end of the renal function studies were fixed with 4% paraformaldehyde in 0.1 M sodium phosphate buffer (pH 7.4) for 24 h at 4°C, placed in 70°C ethanol, and embedded in paraffin. Longitudinal sections 5-µm thick were stained via Gomori's process. The presence of interstitial fibrosis was detected based on the presence of green fibers, and was considered indicative of chronic injury. The tissues were evaluated for glomerular injury and tubulointerstitial fibrosis with a higher power objective (40×) on an Axioskop MOT Plus II Microscope (Carl Zeiss, Jena, Germany) by a blinded observer.

### Immunohistochemical Analysis

The immunohistochemical analysis was used to identify tissue markers of early, middle and later CsA-induced injury. Thus, osteopontin, TNF-α, IL-6 and ED-1 (macrophage antigen) were indicative of leukocyte infiltration and inflammation at all-time points studied. Collagen IV, α-SMA, vimentin and TGF-β were indicative of middle to later lesion.

The primary antibodies used for immunohistochemical analysis of the renal tissue collected at the end of the renal function studies were monoclonal anti-ED1 (macrophage antigen; MCA341R, Serotec, Oxford, United Kingdom); polyclonal anti-TGF-β (sc-7892, Santa Cruz, CA, USA); monoclonal anti-fibronectin (MAB1926, Millipore, CA, USA); monoclonal anti-collagen IV (MAB1910, Millipore, CA, USA); polyclonal anti-osteopontin (AB1870, Millipore, CA, USA); monoclonal anti-IL-6 (840234, R&D, Minneapolis, USA); monoclonal anti-TNF-α (840174, R&D Systems, Minneapolis, USA); monoclonal anti-α-SMA (M0851, Dako, Denmark); and monoclonal anti-vimentin antibody (M0725, Dako, Denmark).

Paraffin-embedded sections (3 µm) were incubated for 20 min with sodium citrate at 96°C. Then, kidney fragments were treated with 3% hydrogen peroxide diluted in methanol for 30 min. The sections were subsequently incubated overnight at 4°C with antibodies against α-SMA (1∶1000), TGF-β (1∶80), collagen IV (1∶250), osteopontin (1∶500), TNF-α (1∶100), IL-6 (1∶100) and fibronectin (1∶100) or at room temperature for 1 hour with anti-ED1 (1∶1000) and anti-vimentin (1∶500) antibodies. Nonspecific protein binding was blocked by simultaneous incubation with 1% bovine serum albumin (BSA) in PBS. Negative controls were incubated with an equivalent concentration of 1% PBS-BSA instead of primary antibody. Subsequently, the sections were incubated for 30 min with a biotinylated secondary antibody followed by avidin-conjugated horseradish peroxidase (K0690 kit, Dako, CA, USA) and then enhanced with diaminobenzidine chromogenic substrate (LSAB detection kit, K3468, Dako, CA, USA) at room temperature. The sections were counterstained with hematoxylin, dehydrated, and mounted. Analysis of the renal cortex was performed with a Zeiss Axioskop 2 (Zeiss, Jena, German) using AxioVision software (Carl Zeiss, Jena, Germany). In the immunostaining for α-SMA, the periglomerular areas were assessed separately. To determine the number of infiltrating macrophages (ED1-positive cells) in the renal cortical tubulointerstitial area, 25 grid fields of the renal cortex were analyzed (Zeiss Axioskop 2). An observer blinded to the treatment groups evaluated all the slides at random.

### Statistical Analysis

The results are presented as the mean ± standard deviation. Comparisons between the CsA and VH groups were performed using Student's *t* test or the Mann-Whitney test, as appropriate. A two-tailed P-value <0.05 was considered statistically significant.

## Results

CsA-treated animals had elevated CsA blood levels and reduced significantly their body weight compared with VH-treated animals. The hematocrit was similar in all groups. These results are summarized in Table 1.

**Table 1:** Weight gain, renal function and hemodynamics, hematocrit and CsA blood levels after 7, 14 or 21 days of daily subcutaneous injection of vehicle (control group, VH) or CsA 15 mg/kg/day (treatment group, CsA).

	Time and Groups					
	Early (7 days)		Mid-Term (14 days)		Late (21 days)	
Parameter	VH	CsA	VH	CsA	VH	CsA
Number of animals/group	8	7	8	8	8	8
Weight gain (g)	20±5	-15±4****	22±13	-29±13****	14±10	-76±25****
Glomerular filtration rate (mL/min/100 g)	0.94±0.15	0.19±0.08****	0.82±0.09	0.18±0.06****	1.00±0.19	0.15±0.05****
Renal blood flow (mL/min)	3.9±1.2	1.5±0.5***	4.3±1.0	0.9±0.5****	2.9±0.8	0.8±0.2****
Microalbuminuria (mg/L)	4.2±1.2	15.1±7.8*	3.8±1.9	3.8±1.5	4.3±0.9	3.9±1.4
Serum creatinine (mg/dL)	0.86±0.29	1.45±0.31**	0.91±0.20	1.45±0.11****	0.91±0.16	1.89±0.49***
Creatinine clearance (mL/min/100 g)	0.23±0.07	0.17±0.06	0.23±0.09	0.13±0.03*	0.18±0.08	0.09±0.03*
Mean arterial pressure (mmHg)	99±16	81±4*	107±16	66±5****	108±10	61±11****
Renal vascular resistance (mmHg/mL/min)	25±6	60±17****	25±5	92±42***	32±7	80±26***
Urinary output (mL/24 h)	15.2±4.5	14.9±8.6	13.4±4.3	12.9±3.1	8.9±4.2	12.8±3.9
Urinary osmolality	289±67	150±91*	242±70	139±83*	341±126	167±41*
Sodium excretion fraction (%)	0.61±0.17	0.96±0.41*	0.43±0.21	1.46±0.24****	0.46±0.08	1.74±0.80**
Potassium excretion fraction (%)	5.7±3.3	6.6±2.7	5.7±3.3	8.3±13.1	4.5±3.2	16.3±14.2*
Hematocrit (%)	49±5	49±2	46±2	45±2	45±3	44±4
Blood CsA (ng/mL)	-	256±51	-	512±23	-	599±121

The data are presented as the mean ± SD.

* p<0.05,

** p<0.01,

*** p<0.001 and

**** p<0.0001 (CsA versus VH).

### Renal Function in the Treatment and Control Groups

CsA significantly reduced the GFR and the RBF and significantly increased renal vascular resistance when compared with VH at all time points analyzed. Creatinine clearance was significantly reduced in the CsA treatment groups after 14 and 21 days of treatment (Table 1). Urinary output did not differ among the groups at any studied time point. CsA-treated animals had significantly lower urinary osmolality after 7, 14 and 21 days of treatment. The fractional excretion of potassium was significantly increased in the CsA group compared with the VH group after 21 days of treatment (Table 1). All studied groups presented extremely low urinary sodium levels and urinary osmolality due to the low-salt diet. These results confirmed the efficacy of the intervention in generating the groups for comparison in the present study.

### Early Injury (7 days)

#### Urinary Biomarkers

**Figure 1:**
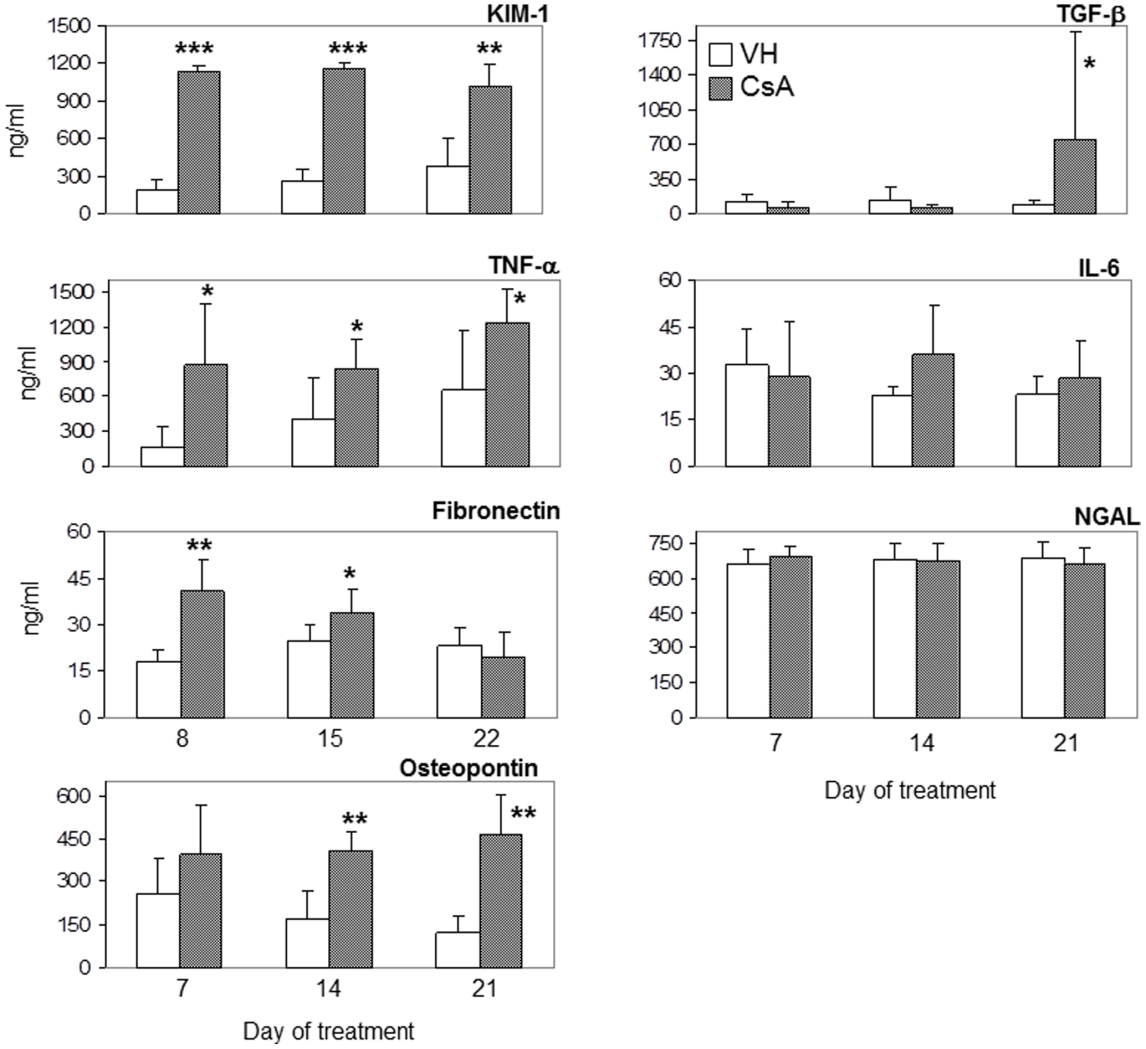
Urinary biomarker levels. Urinary ELISA assays (ng/mL) after 7, 14 or 21 days of treatment with vehicle (VH) or 15 mg/kg cyclosporine (CsA). The data are presented as the mean ± SD. * p<0.05, ** p<0.01, *** p<0.001 and **** p<0.0001 (CsA versus VH).

After 7 days the microalbuminuria, urinary KIM-1, TNF-α, and fibronectin were significantly increased in CsA-treated animals when compared with VH-treated rats (Table 1 and Figure 1). When compared to controls, microalbuminuria increased 3.6 times, KIM-1 6.2 times, TNF-α 5.4 times and fibronectin 2.3 times.

#### Renal Tissue Histochemistry and Immunohistochemistry

**Figure 2:**
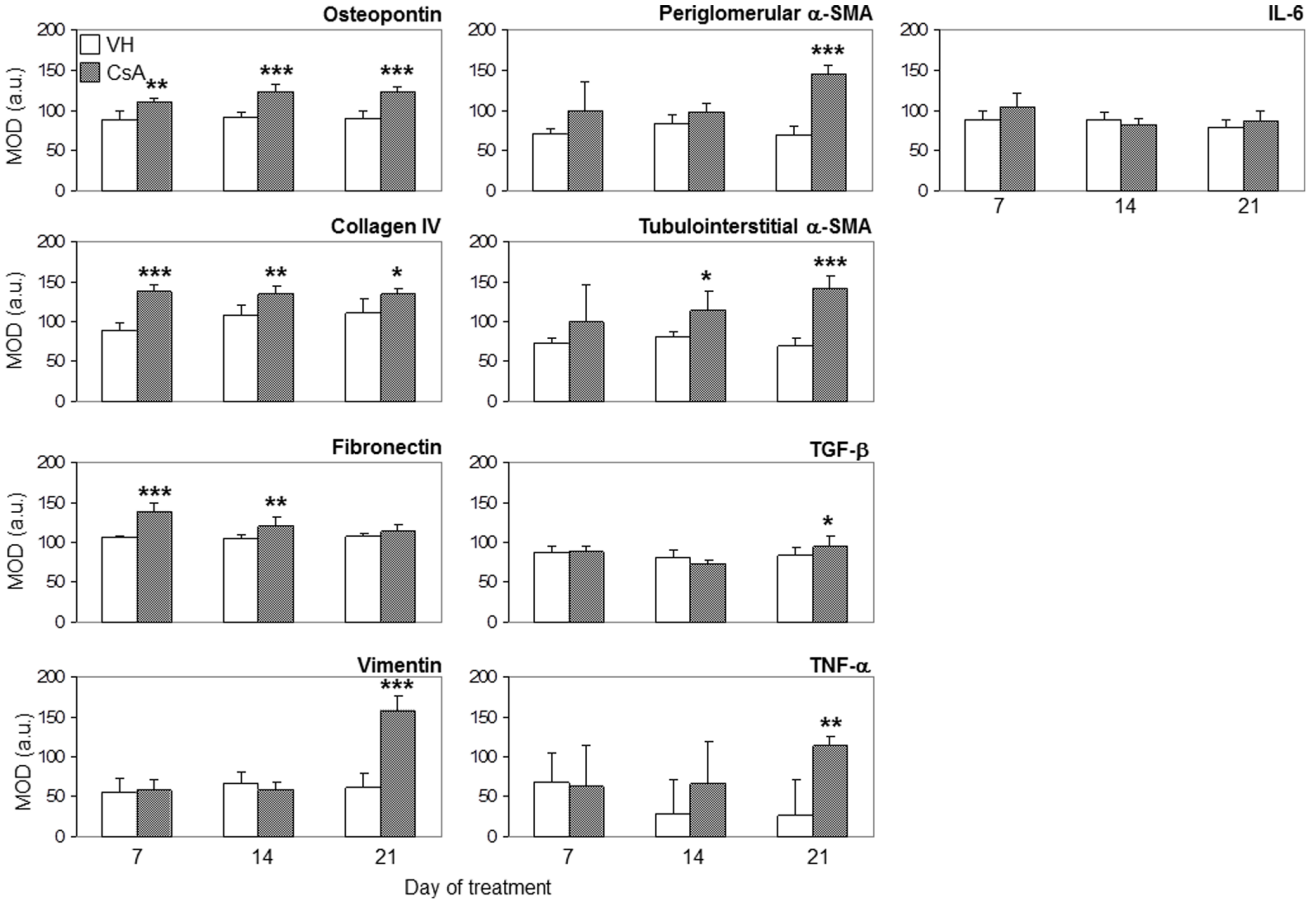
Immunohistochemical detection of injury markers in renal cortex tissue. Mean optical densitometry, MOD, arbitrary units after 7, 14 or 21 days of treatment with vehicle (VH) or 15 mg/kg cyclosporine (CsA). The data are presented as the mean ± SD. * p<0.05, ** p<0.01 and *** p<0.001 (CsA versus VH).

**Figure 3:**
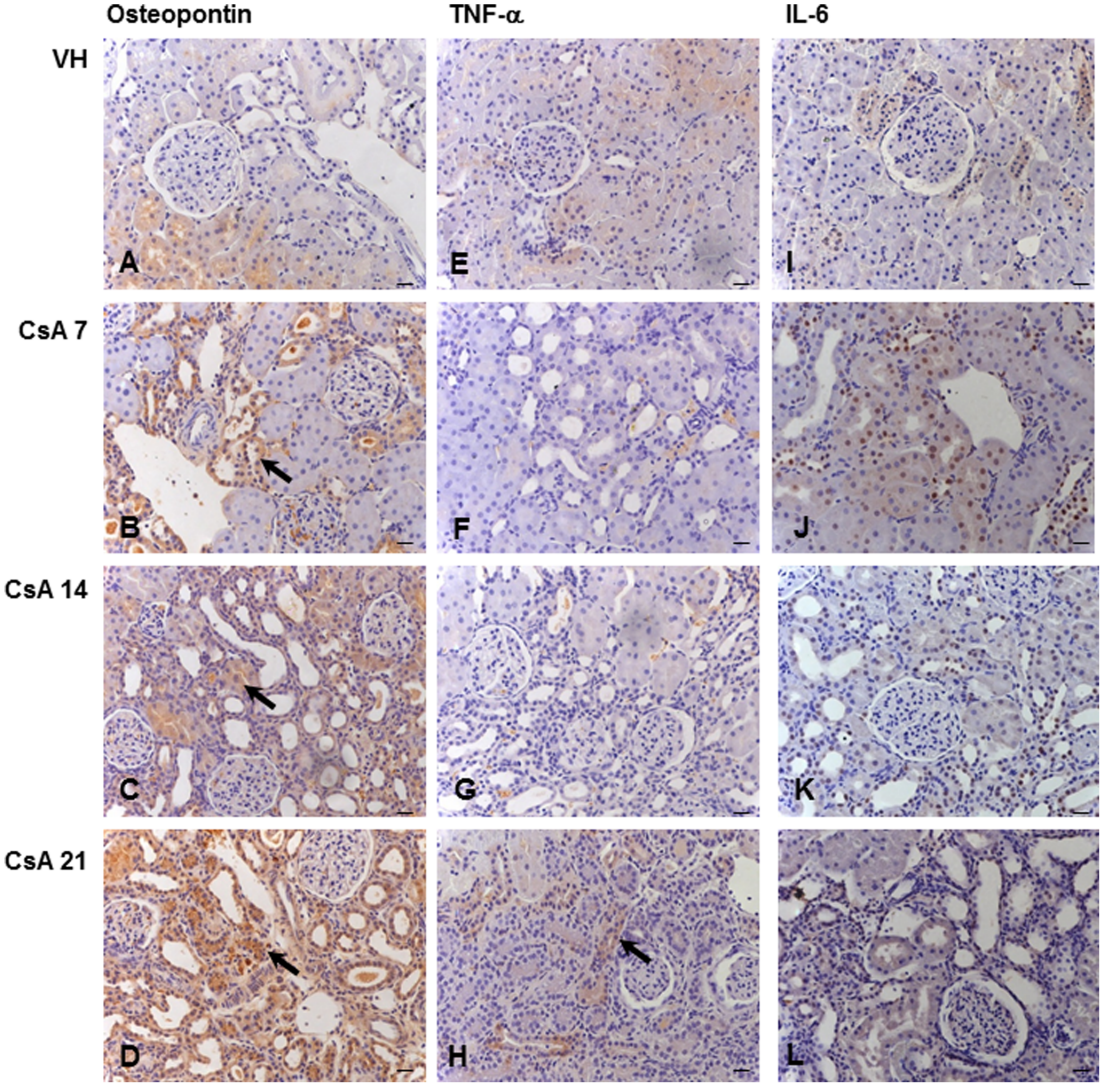
Kidney immunolocalization of tubulointerstitial osteopontin, TNF-α and IL-6. Osteopontin (A–D), TNF-α (E–H) and IL-6 (I–L) after 7, 14 or 21 days of treatment with vehicle (VH) or 15 mg/kg cyclosporine (CsA). Black arrows indicate renal inflammation, p≤0.01 (B–D and H). Hematoxylin counterstain. Bars = 10 µm.

**Figure 4:**
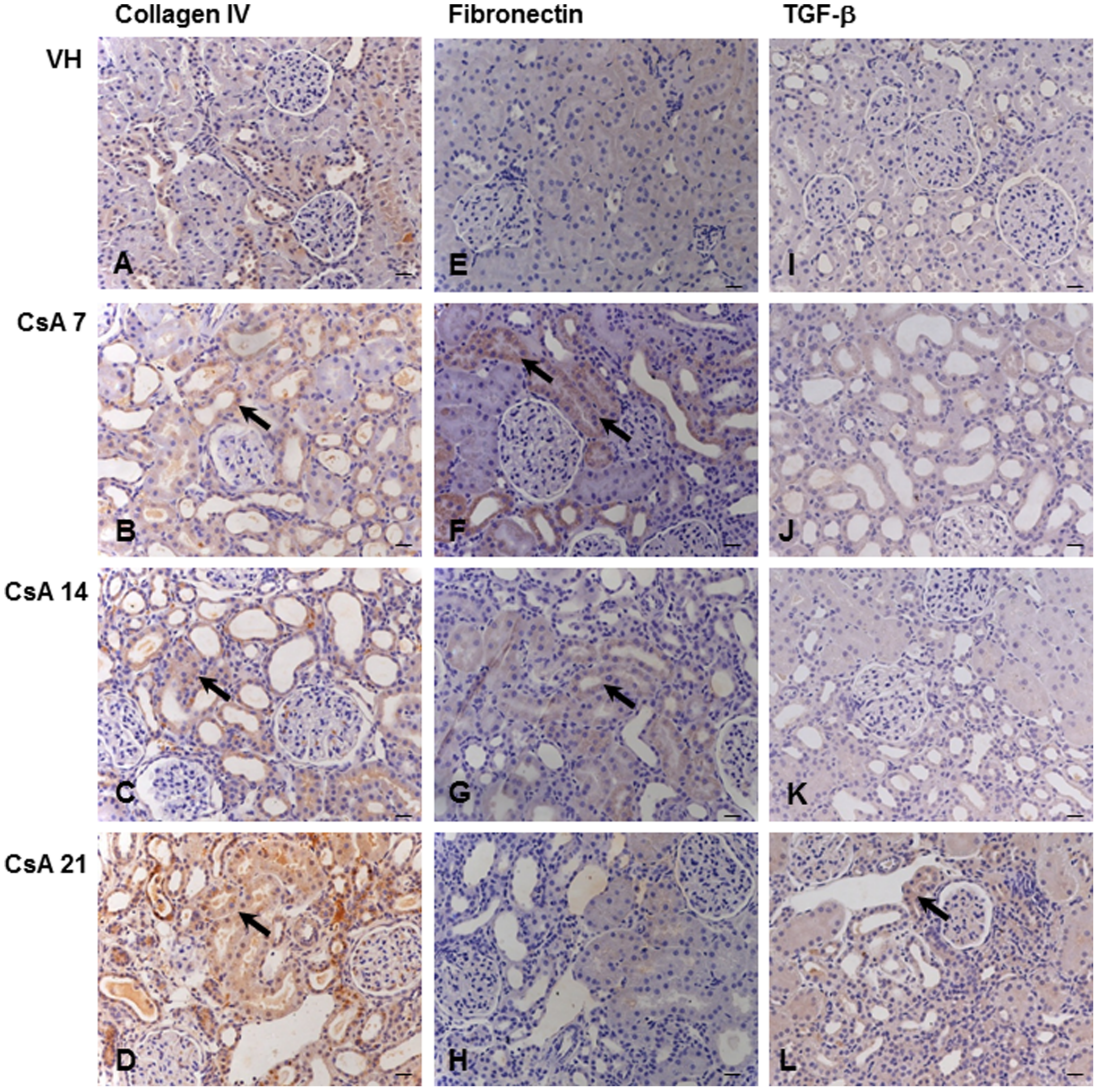
Immunolocalization of tubulointerstitial collagen IV, fibronectin and TGF-β. Collagen IV (A–D), fibronectin (E–H) and TGF-β (I–L) after 7, 14 or 21 days of treatment with vehicle (VH) or 15 mg/kg cyclosporine (CsA). Black arrows indicate renal inflammation and injury, p≤0.05 (B–D, F–G and L). Hematoxylin counterstain. Bars = 10 µm.

After one week, the renal expression of osteopontin (Figure 2 and Figure 3B), collagen IV (Figure 2 and Figure 4B) and fibronectin (Figure 2 and Figure 4F) was significantly upregulated in CsA-treated rats compared with the control group.

### Mid-Term Injury (14 days)

#### Urinary Biomarkers

After two weeks, microalbuminuria in the treatment group decreased to values similar to those in the control group. Urinary KIM-1, TNF-α, and fibronectin remained significantly increased in CsA-treated animals (4.4, 2.1 and 1.3 times, respectively). At this time point, urinary osteopontin increased significantly (1.6 times) in CsA-treated rats (Table 1 and Figure 1).

#### Renal Tissue Histochemistry and Immunohistochemistry

**Figure 5:**
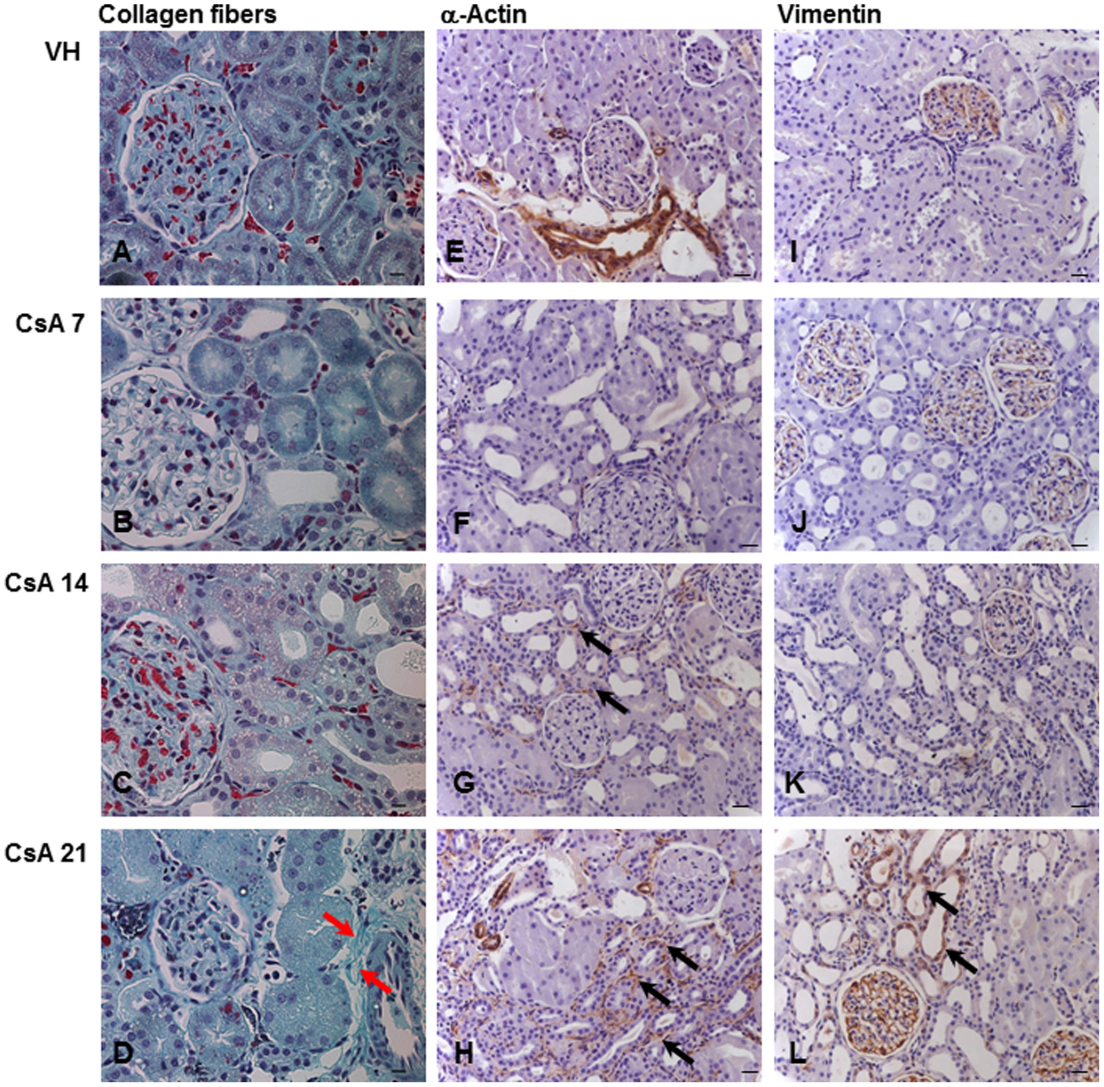
Kidney injury induced by CsA. Collagen fibers stained green by Gomori's method (A–D). Red arrows indicate fibrosis (D). Immunolocalization of tubulointerstitial α-SMA (E–H) and vimentin (I–L) after 7, 14 or 21 days of treatment with vehicle (VH) or 15 mg/kg cyclosporine (CsA). Black arrows indicate renal injury, p≤0.001 (G–H and L). Hematoxylin counterstain. Bars = 10 µm.

**Figure 6:**
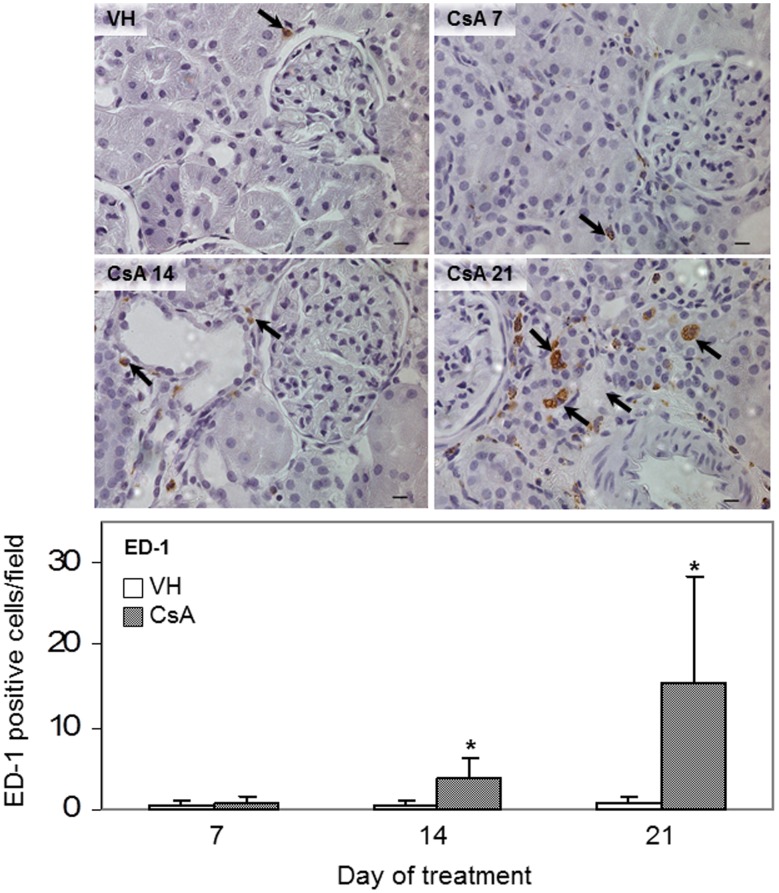
Macrophage infiltration. Immunohistochemistry by ED-1 immunostaining (arrows) after 7, 14 or 21 days of treatment with vehicle (VH) or 15 mg/kg cyclosporine (CsA). The data are presented as the mean ± SD. * p<0.05 (CsA versus VH). Hematoxylin counterstain. Bars = 10 µm.

The renal expression of osteopontin (Figure 2 and Figure 3C), collagen IV (Figure 2 and Figure 4C) and fibronectin (Figure 2 and Figure 4G) remained significantly upregulated in rats treated with CsA after 14 days. At this time point, tubulointerstitial α-SMA (Figure 2 and Figure 5G) was increased significantly in CsA-treated animals. After two weeks, the number of renal macrophages was significantly higher in CsA-treated rats (Figure 6) than in control rats.

### Late Injury (21 days)

#### Urinary Biomarkers

After three weeks, urinary KIM-1 and TNF-α were still increased (respectively, 2.7 and 1.9 times) in the CsA group, but urinary fibronectin decreased to a value similar to that in the control group. Urinary osteopontin remained significantly increased in CsA-treated animals (2.4 times). At this time point, urinary TGF-β increased significantly (8.5 times) in the CsA-treated animals when compared with the control group (Figure 1).

#### Renal Tissue Histochemistry and Immunohistochemistry

After three weeks, the renal expression of osteopontin (Figure 2 and Figure 3D), collagen IV (Figure 2 and Figure 4D), and tubulointerstitial α-SMA (Figure 2 and Figure 5H) remained significantly upregulated, and significantly more renal macrophages (Figure 6) were present in CsA-treated rats compared to control rats. At this time point, the renal expression of fibronectin was decreased and similar to that in the control group (Figure 2 and Figure 4H). After three weeks, the renal expression of vimentin (Figure 2 and Figure 5L) and TNF-α (Figure 2 and Figure 3H) was increased in CsA-treated animals compared with the control group. Renal tissue TGF-β was significantly overexpressed only after 21 days of CsA treatment (Figure 2 and Figure 4L).

None of the studied animals had detectable levels of podocin in their urine. There were no significant differences in the levels of urinary IL-6 or NGAL between CsA-treated and control group animals at any time point.

IL-6 renal expression was similar in CsA and control animals at all studied time points (Figure 2 and Figure 3, J–L).

### Histopathological Assessment of Renal Damage

Tubulointerstitial fibrosis (Figure 5, A–D) was only observed in rats treated with CsA for 21 days (Figure 5, D). There was no detectable glomerular injury in CsA- or VH-treated animals at any time point.

## Discussion

Salt-depleted rats were studied after one, two and three weeks of CsA or vehicle treatment, according to a well-established CsA nephrotoxicity model, to assess potential urinary biomarkers for the identification of different stages of CsA-induced kidney injury. The functional and structural changes found in CsA-treated animals in the present study are consistent with previously published results that utilized the same model of CsA nephrotoxicity.

The most interesting finding of this study is that increased levels of urinary KIM-1, TNF-α, fibronectin, osteopontin and TGF-β identified the different stages of CsA-induced nephrotoxicity, correlating consistently with tissue cytokine expression and the functional and structural kidney changes caused by CsA. If confirmed in clinical studies, these findings might allow the safer and more rational use of CsA in transplant and in autoimmune diseases patients.

### Early injury (7 days)

KIM-1, TNF-α, microalbuminuria and fibronectin were significantly elevated urinary biomarkers in CsA-treated animals at the early time point, indicating acute nephrotoxicity. At this time point, the GFR was already impaired, but renal fibrosis was undetectable. After 7 days of CsA treatment, the renal expression of osteopontin, collagen IV and fibronectin was upregulated, suggesting that the mechanism that controls the later development of interstitial fibrosis was precociously activated in the course of CsA nephrotoxicity.

KIM-1 is an undetectable transmembrane glycoprotein in normal kidneys, which sheds into the urine after proximal tubular kidney injury. It has been consistently demonstrated as an early indicator of kidney injury in both rodents and humans [5], [7], [22] and is considered as a highly sensitive and specific urinary biomarker for monitoring drug-induced kidney injury [23]. KIM-1 was significantly increased at all stages of CsA-induced nephrotoxicity in the current study, suggesting that CsA-induced tubular injury occurs early, preceding interstitial fibrosis, and is a continuous process during the period of drug exposure.

TNF-α is a pleiotropic proinflammatory and immunoregulatory cytokine that acts as a mediator of tissue injury. In the kidney, infiltrating macrophages and endothelial, mesangial, glomerular, and tubular epithelial cells synthesize and release TNF-α. This cytokine is a key participant in the pathogenesis of kidney injury, triggering inflammation, apoptosis, and the accumulation of extracellular matrix, impairing glomerular blood flow and damaging the glomerular permeability barrier with the development of albuminuria [24]. Urinary TNF-α increased early after ischemia-reperfusion injury in pigs [25]. In the current study, the early increase in urinary TNF-α likely resulted from the direct action of CsA on resident renal cells or from tubular cells that suffered ischemia due to CsA-induced pre-glomerular vasoconstriction.

Fibronectin is a ubiquitous kidney matrix glycoprotein that connects epithelial cells via integrin to the extracellular matrix collagen [26]–[27]. Its overexpression occurs in experimental and clinical acute tubular injury [44]–[45] and in models of CsA-induced nephrotoxicity [28]–[29]. The early increase in the urinary excretion and renal tissue overexpression of fibronectin observed in this study were most likely associated with tubular injury resulting directly from CsA toxicity to renal tubular cells or CsA-induced renal ischemia.

### Mid-term injury (14 days)

After 14 days of CsA treatment, the urinary excretion of KIM-1, TNF-α and fibronectin remained significantly elevated, and that of urinary osteopontin, increased significantly. At this midpoint, the renal tissue expression of osteopontin, collagen IV and fibronectin remained elevated, there was significant renal macrophage infiltration, and the kidney tubulointerstitial expression of α-SMA, a microfilament that marks epithelial-mesenchymal transition and fibroblast activation, increased significantly. These data indicated a transition from acute to chronic CsA-induced nephrotoxicity.

Osteopontin is a monocyte-macrophage adhesive protein produced primarily by the distal nephron [30]–[31] and secreted into urine [32] in the normal kidney. It attracts macrophages and participates in the development of renal fibrosis after kidney injury [14], [33]. Our results are consistent with previous data indicating that osteopontin contributes to chronic CsA-induced nephrotoxicity by recruiting macrophages, triggering epithelial-mesenchymal transition in renal tubular epithelial cells and promoting tubulointerstitial fibrosis [11], [34].

The elevated urinary KIM-1 at this point of the study was likely associated with fibrosis development. Renal interstitial fibrosis and chronic inflammation were already correlated with the elevated tissue expression and urinary release of KIM-1 [23], [35]. In a consistent way, treating salt-depleted rats with CsA for 18 days increased urinary KIM-1 excretion and promoted interstitial fibrosis (53). Urinary TNF-α levels remained elevated at the midpoint of the CsA treatment course in the current study. TNF-α is elevated during chronic inflammatory states, where it participates in the mechanisms controlling progressive renal damage by recruiting immune cells to the kidneys, and via direct effects on renal cells [36]. The increased urinary excretion and renal tissue expression of fibronectin observed at the midpoint of this study are consistent with the progression of chronic kidney injury. Increased urinary fibronectin correlates with the progression of renal dysfunction in primary glomerulopathies [37], and elevated renal expression of fibronectin was associated with interstitial fibrosis development [38].

### Late injury (21 days)

Three weeks of CsA treatment caused overt CsA-induced chronic nephrotoxicity characterized by interstitial fibrosis. Urinary KIM-1 and osteopontin levels remained significantly elevated, urinary TNF-α peaked, most likely because of increased renal macrophage infiltration, and urinary TGF-β increased significantly for the first time in this study. Likewise, the renal expression of TGF-β increased significantly, the number of renal macrophages peaked, and accordingly, the renal expression of TNF-α significantly increased, indicating the progression of kidney chronic injury. CsA treatment for 21 days also significantly increased the renal expression of vimentin, a marker of proximal tubular cell injury and epithelial-mesenchymal transition. CsA-induced upregulation of vimentin expression in the renal tubule and interstitial area was previously observed in chronic CsA nephrotoxicity [39]–[42]. Consistently, the tubulointerstitial expression of α-SMA, collagen IV and osteopontin remained elevated, and the periglomerular expression of α-SMA increased significantly.

TGF-β, which is released by macrophages, is involved in the development of CsA-induced interstitial fibrosis [11], [43]–[47] and is related to the epithelial-mesenchymal transition process [29], [42], [48]–[51].

The combination of increased urinary KIM-1, osteopontin and TGF-β in this study indicated the development of chronic CsA nephrotoxicity.

### Study Limitations

This was an animal model study, and thus, confirmation of these results by clinical studies is necessary before the possible use of these biomarkers in predicting CsA nephrotoxicity.

CsA-treated rats under low salt diet disclosed significantly lower BP values, particularly after 3 weeks of treatment. However, most of the observed changes in the urinary biomarkers (KIM-1, TNF-α, fibronectin, microalbuminuria) occurred after 7 days of treatment, when the intraarterial BP of CsA-treated rats was 81±4 mmHg. Only osteopontin and TGF-β, elevated when the BP decrease was more marked. Those biomarkers are associated to development of renal fibrosis, and it is improbable that their changes were due to the observed BP variation.

## Conclusions

Urinary KIM-1, TNF-α, fibronectin, microalbuminuria, osteopontin and TGF-β were reliable biomarkers of CsA nephrotoxicity, correlating with the development of CsA-induced renal functional and structural injury and the temporal course of CsA nephrotoxicity from acute to chronic.

The clinical implications of these findings are evident. If proved efficacious in clinical studies, these biomarkers will allow the safer and more rational use of CsA.
